# Utilization of Artificial Intelligence Coupled with a High-Throughput, High-Content Platform in the Exploration of Neurodevelopmental Toxicity of Individual and Combined PFAS

**DOI:** 10.3390/jox15010024

**Published:** 2025-02-02

**Authors:** Seth D. Currie, David Blake Benson, Zhong-Ru Xie, Jia-Sheng Wang, Lili Tang

**Affiliations:** 1 Interdisciplinary Toxicology Program, University of Georgia, Athens, GA 30602, USAdavid.benson@uga.edu (D.B.B.);; 2 Department of Environmental Health Science, College of Public Health, University of Georgia, Athens, GA 30602, USA; 3 School of Electrical and Computer Engineering, College of Engineering, University of Georgia, Athens, GA 30602, USA

**Keywords:** per- and polyfluoroalkyl substances, *Caenorhabditis elegans*, neuron development, artificial intelligence

## Abstract

Per- and polyfluoroalkyl substances (PFAS) are synthetic chemicals used in various products, such as firefighting foams and non-stick cookware, due to their resistance to heat and degradation. However, these same properties make them persistent in the environment and human body, raising public health concerns. This study selected eleven PFAS commonly found in drinking water and exposed Caenorhabditis elegans to concentrations ranging from 0.1 to 200 µM to assess neurodevelopmental toxicity using a high-throughput, high-content screening (HTS) platform coupled with artificial intelligence for image analysis. Our findings showed that PFAS such as 6:2 FTS, HFPO-DA, PFBA, PFBS, PFHxA, and PFOS inhibited dopaminergic neuron activity, with fluorescence intensity reductions observed across concentrations from 0.1 to 100 µM. PFOS and PFBS also disrupted synaptic transmission, causing reduced motility and increased paralysis in aldicarb-induced assays, with the most pronounced effects at higher concentrations. These impairments in both neuron activity and synaptic function led to behavioral deficits. Notably, PFOS was one of the most toxic PFAS, affecting multiple neurodevelopmental endpoints. These results emphasize the developmental risks of PFAS exposure, highlighting the impact of both individual compounds and mixtures on neurodevelopment. This knowledge is essential for assessing PFAS-related health risks and informing mitigation strategies.

## 1. Introduction

Per- and polyfluoroalkyl substances (PFAS) represent a broad category of human-made fluorinated compounds that are prevalent environmental contaminants, leading to frequent human exposure [1]. These compounds are highly fluorinated aliphatic substances where all hydrogen atoms attached to one or more carbon atoms have been replaced by fluorine atoms [2]. The robust carbon-fluoride bonds in PFAS contribute to their exceptional stability [3]. This stability makes them resistant to degradation by high temperatures, natural light, chemical reactions, and biological processes [4]. Toxicologists have deemed PFAS as ‘forever chemicals’ due to their high stability. Many PFAS are exploited for their surfactant characteristics, leading to their intentional inclusion in a wide array of products such as cookware, packaging materials, clothing, carpets, and foams used in firefighting [5]. Biomonitoring studies have shown that PFAS are found in the blood over 98% of humans within the United States [6,7]. PFAS exposure occurs through multiple channels, such as consuming food and water, breathing air, using personal care products, and coming into contact with common items like outerwear, rugs, cleaning supplies, paper products, and indoor dust [8,9,10].

Epidemiological evidence links PFAS to a broad spectrum of health concerns, including thyroid dysfunction [11,12], immune system impairments [13,14,15], liver disease and cancer [16], disturbances in lipid and insulin levels [17,18], kidney disease and cancer [19,20], reproductive and developmental issues [21,22], and impacts on neurodevelopment [23,24,25]. Among these, developmental neurotoxicity (DNT) from PFAS exposure is a significant concern, as it has been associated with neurodevelopmental disorders, including autism spectrum disorder (ASD), attention-deficit/hyperactivity disorder (ADHD), and intellectual disabilities [26]. PFAS chemicals disrupt critical processes in brain development, such as synaptic formation, neurotransmission, and neural connectivity [27]. The mechanisms underlying these effects include oxidative stress, mitochondrial dysfunction, inflammation, and disruption of the blood–brain barrier, all of which compromise neuronal integrity and brain function [28]. These findings highlight the need for the development of more effective models to better understand PFAS’s impact on neurodevelopment and investigate its effects on neuronal processes.

*Caenorhabditis elegans* (*C. elegans*) has emerged as a powerful model organism for studying neurotoxicity, particularly due to its well-known and rapid developmental cycle. Its utility is particularly evident when investigating the impacts of PFAS on dopaminergic neurons and overall neuronal health. These small, transparent nematodes offer numerous advantages for toxicological studies, including a short lifespan, ease of cultivation, and the ability to produce many offsprings, making them an excellent model for laboratory research [29]. The optical transparency of *C. elegans* provides a unique advantage for researchers, allowing them to examine neuronal cells and features without dissection, which is further enhanced by the use of fluorescent protein reporters to monitor changes in neuronal morphology and function in real time [30]. Importantly, nematodes possess homologous genes for approximately 80% of those found in humans [31]. This strong conservation makes *C. elegans* an ideal platform for evaluating the neurotoxic effects of PFAS, which are known to impair neuronal structure, disrupt synaptic connectivity, and cause deficits in neurobehavioral responses. In particular, the well-characterized dopaminergic system of *C. elegans* has been well-characterized, making it a suitable model for studying the impacts of environmental exposures on DA neurons [32,33]. Furthermore, the disruption of dopaminergic function in C. elegans exposed to PFAS has direct consequences on behavior, particularly in tasks that require learning and memory [34,35].

The vast number of PFAS, along with their structural diversity, presents a significant challenge for traditional toxicity testing approaches. With thousands of PFAS compounds in the environment, many of which lack comprehensive toxicity data, there is an urgent need for high-throughput, high-content platforms capable of efficiently assessing the neurotoxic potential of these chemicals. These platforms enable the rapid evaluation of neurotoxic endpoints, such as changes in neuronal morphology, synaptic function, and behavior, at a scale and speed that traditional methods cannot achieve [36]. Using the *C. elegans* model, our laboratory has created high-throughput screening platforms to evaluate the toxicity of PFAS and various neurotoxins [35,37,38]. To maximize the effectiveness of these platforms, artificial intelligence (AI) tools are increasingly being integrated to enhance data analysis. AI-driven approaches allow for the processing of large datasets, identification of subtle neurotoxic effects, and automated assessments, thus reducing analysis time and improving consistency [39]. By enabling advanced image analysis and pattern recognition, AI technologies support faster identification of hazardous PFAS, facilitating more efficient regulatory decision-making and risk assessment.

The integration of artificial intelligence (AI) in toxicological research offers a novel approach to streamline data analysis and improve the accuracy and efficiency of assessing chemical toxicity. AI encompasses the creation of computer systems designed to perform tasks traditionally requiring human intelligence [40]. These tasks include recognizing patterns, learning from data, and making decisions. By leveraging advanced algorithms and machine learning techniques, AI systems can process vast amounts of information, identify trends, and adapt to new data. The advancements in AI and the life sciences are deeply interconnected. AI technologies, such as machine learning and data analytics, are revolutionizing the life sciences by enabling more efficient data processing, complex pattern recognition, and predictive modeling [41]. Neuroscience has been significantly accelerated with the utilization of AI [42]. AI techniques have revolutionized the analysis of neuroimaging datasets, offering significant potential to expedite connectomic analysis [43]. This technology can be applied to investigate the impacts of PFAS on developing dopaminergic neurons through morphological alterations, synaptic formation, and neuronal activity in *C. elegans*.

Deep learning, a subset of machine learning, has been particularly transformative, utilizing artificial neural networks inspired by the structure and functioning of the human brain [44]. These networks consist of multiple stacked layers, allowing AI systems to interpret complex, high-dimensional data and extract intricate patterns across diverse fields such as computer vision, language processing, and biomedicine [45]. This layered approach has accelerated advances in life sciences by facilitating detailed image analysis, disease prediction, and drug discovery, enabling scientists to make strides in understanding human health and biology at unprecedented scales [46]. Deep learning’s impact on neuroimaging is profound, as algorithms designed for massive data analysis allow researchers to identify subtle neural pathways and interactions between neurons, bringing new insights into neurodegenerative diseases and cognitive functions [47]. The adaptability of deep learning models is especially advantageous for studying environmental effects on neural development. By enhancing our analytical capabilities, deep learning technologies not only deepen our understanding of life sciences and neuroscience but also create actionable insights that support efforts in environmental safety and public health, illustrating the profound synergy between AI and scientific discovery.

In this study, eleven PFAS were selected according to their widespread occurrence in different water bodies [48], which represent a wide range of typical PFAS structures, including perfluoroalkyl carboxylic acids (perfluorobutanoic acid [PFBA], perfluorohexanoic acid [PFHxA], perfluorooctanoic acid [PFOA], perfluorononanoic acid [PFNA]), sulfonic acids (perfluorobutanesulfonic acid [PFBS], perfluorohexanesulphonic acid [PFHxS], perfluorooctanesulfonic acid [PFOS]), sulfonamides and derivatives (perfluorooctanesulfonamide [PFOSA], [NetFOSAA]), fluorotelomers (6:2 fluorotelomer sulfonic acid [6:2 FTS]), and new substitutes (hexafluoropropylene oxide dimer acid [HFPO-DA], the acid form of GenX). The goal is to investigate the impact of PFAS exposure on dopaminergic (DA) neurons in *C. elegans*, with a specific focus on morphological alterations, synaptic formation, and behavior (neuron activity). By focusing on these aspects of dopaminergic neuron development, we aim to understand the mechanisms through which PFAS affect neurodevelopment. Additionally, this study utilized AI tools to enhance data analysis, providing fast and accurate results that will facilitate high-throughput screening of PFAS toxicity. Through these combined approaches, this study contributes valuable insights into the neurotoxic effects of PFAS and helps improve methodologies for assessing the environmental and health risks of these contaminants.

## 2. Materials and Methods

### 2.1. Chemicals

A review of data from 23 studies investigated the prevalence of PFAS in various water bodies to determine the most common PFAS [48]. The analysis also highlighted the primary PFAS in each category and identified several alternatives used to replace legacy PFAS. The 10 PFAS chosen for this study were PFBA (95%, BCCF2984, COA), PFHxA (98%, P102-28684, COA), PFOA (95%, WXBD6815, COA), PFNA (97%, 394459, COA), PFBS (98%, P151-08994, COA), PFHxS (95%, 751400, COA), PFOS (98%, 830800, COA), NEtFOSAA (95%, P102-28720, COA), 6:2 FTS (95%, 754100, COA), PFOSA (96%, CDS010729, COA), and HFPO-DA (96%, 00022309, COA).

Analytical grade PFBA, PFNA, and PFOA were sourced from Sigma Aldrich (St. Louis, MO). NEtFOSAA, PFHxA, and PFBS were obtained from Astatech Inc. (Bristol, PA, USA). PFOS, PFHxS, HFPO-DA, 6:2 FTS, and PFOSA were procured from Synquest Laboratories, Inc. (Alachua, FL, USA). Stock solutions at a concentration of 1 M were prepared using dimethyl sulfoxide (DMSO), and working solutions were further diluted with K-medium (32 mM KCl and 51 mM NaCl) supplemented with OP50 as a 1 mg/mL food source, resulting in a final DMSO concentration of 0.1% [49].

### 2.2. Mixture Selection

We identified five PFAS that predominantly contribute to the PFAS load in U.S. water sources [50]. From these chemicals, we created a reference mixture based on their relative concentrations in the overall mixture. The estimated proportions were PFOS (30%), PFBA (20%), PFOA (20%), PFHxS (15%), and PFBS (15%). This mixture will serve as a model for typical daily exposure in the U.S. While the total concentration of the mixture may vary, the proportions will remain fixed. Evaluating mixture profile patterns helps in assessing specific PFAS compounds that are frequently found at higher concentrations [51].

### 2.3. C. elegans and Exposure

BZ555 nematodes (dat-1p::GFP) and the *Escherichia coli* strain OP50 were sourced from the Caenorhabditis Genetics Center (Minneapolis, MN, USA). The *C. elegans* were cultivated at 25 °C on nematode growth medium (NGM) plates with OP50 as their food. To obtain synchronized L1 larvae, we performed alkaline lysis (10M NaOH, 2% sodium hypochlorite), followed by overnight hatching in k-medium [49]. For individual neuron imaging experiments, exposure concentrations were set at 100, 1, and 0 μmol/L, while for synaptogenesis studies, the concentrations were 200, 100, 10, 1, and 0.1 μmol/L. These concentrations were chosen based on our previous findings [35] and the U.S. EPA’s ToxCast program recommendations for in vivo assays [52]. Considering *C. elegans* is an in vivo model with interspecies variations, we selected 200 μmol/L as the highest concentration to effectively observe the elicited effects and responses.

### 2.4. High-Throughput, High-Content Platform for Dopaminergic Neurons in Nematodes

As previously described, a high-throughput platform was utilized using the COPAS BIOSORT (Union Biometrica, Inc., Holliston, MA, USA) to investigate the impacts of PFAS on dopaminergic neurons [35,37,53]. Nematode worms were sorted and distributed into 96-well plates by the COPAS BIOSORT, with each well filled with k-medium, a bacterial food source, and varying levels of PFAS. The experiments were conducted in triplicate, with three separate plates, each containing 10 technical replicates per exposure level. Negative controls were included on each plate. The plates were incubated at 25 °C with continuous shaking, and assessments were conducted at both 24 and 48 h. At the time of assessment, the vivoChip-2x technology (vivoVerse, TX, USA) was employed to analyze the impacts of PFAS on individual dopaminergic neurons, providing high-resolution data on their physiological changes [54,55]. Moreover, the Cytation 5 Cell Imaging Multi-Mode Reader (Agilent, CA, USA) was used to capture detailed images of the nematodes, allowing for precise visual documentation and analysis of their condition from exposure. Images were captured using a Sony CMOS, 16-bit color camera (SONY, Tokyo, Japan) at 60× magnification. Integrating the vivoChip-2x and the Cytation imaging system creates a high-throughput, high-content platform for investigating the effects of potential neurotoxins. Data were read, processed, and plotted using GraphPad Prism (Graphpad Software, LA Jolla, CA, USA, Version 10.1.2).

### 2.5. Artificial Intelligence

**Figure 1:**
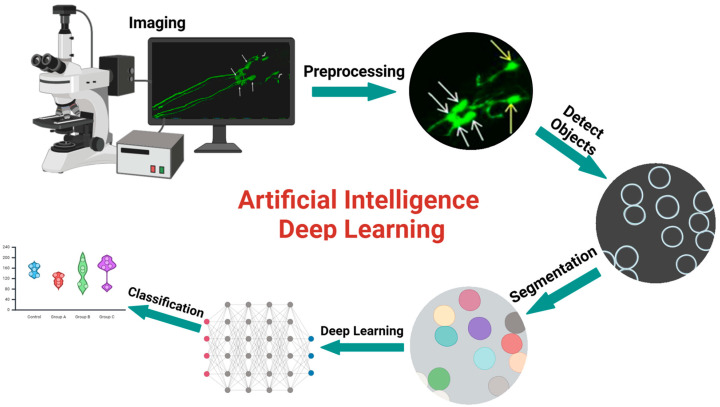
Deep learning image analysis workflow. Image analysis utilizes preprocessing, object detection, segmentation, and classification to determine key features and patterns within the data.

Images were analyzed using DeepImageJ, an open-source framework that integrates deep learning models for image analysis within the ImageJ ecosystem. This innovative tool allows for the seamless incorporation of advanced machine learning techniques into traditional image analysis workflows, enhancing both accuracy and efficiency. For this study, we employed the HPA cell segmentation model, specifically designed for identifying and quantifying cellular structures within complex biological images. This is accomplished through the analysis of both neuronal size and fluorescence intensity. Prior to analysis, all images underwent preprocessing to standardize dimensions, formats, and contrast levels, ensuring optimal input for the model. After importing the images into DeepImageJ, the HPA cell segmentation model was applied, utilizing a convolutional neural network (CNN) trained on an extensive dataset of annotated cellular images. The workflow for the image analysis is outlined in Figure 1. This model excels at accurately segmenting cells based on their morphological characteristics, thus enabling detailed quantification of cellular parameters such as size, shape, and density. To validate the accuracy of the segmentation results, we conducted a series of performance assessments, comparing the model outputs with manually annotated reference images and through traditional fluorescence analysis. Significant changes in morphological structure could indicate that exposure to PFAS impacts neurodevelopment, reflecting disruptions in cellular processes or alterations in neuronal development.

### 2.6. Synaptogenesis Assay

Fifty age-synchronized *C. elegans* L1-stage worms were allocated into individual wells containing 90 μL of testing solution, which consisted of k-medium, a bacterial food source, and varying concentrations of PFAS. The plates were incubated at 25 °C for periods of 24 and 48 h. The experiments were conducted in triplicate, with three separate plates, each containing 10 technical replicates per exposure level. Negative controls were included on each plate. After incubation, the impacts on synaptogenesis were assessed using an aldicarb-sensitive assay to determine the time to paralysis upon aldicarb exposure [56]. Aldicarb (Toronto Research Chemicals, 98%, X11437815, COA) was added into each well with a final concentration of 500 μM. Paralysis was measured with the WMicrotracker-One™ (PhylumTech, Santa Fe, AR, USA, version 2.1) over a 3 h period, with motility loss monitored at fifteen-minute intervals. The aldicarb-induced paralysis assay is a quick and simple method to evaluate alterations in synaptic transmission in *C. elegans* by correlating the rate of neurotransmitter release with the onset of paralysis [57].

### 2.7. Area Under the Curve (AUC)

The area under the curve (AUC) is a quantitative measure that represents the total cumulative effect of a variable over a specified range, capturing the overall magnitude of response or activity in a given dataset [58]. The area under the curve for synaptogenesis was meticulously calculated using the “pracma” package in R to measure the cumulative synaptic response to varying concentrations of test substances, integrating time-course data of synaptic activity to capture the dynamics of synaptic development over time. Higher AUC values indicate a more pronounced synaptic response, reflecting enhanced synaptogenesis, while lower values suggest reduced activity. To facilitate accurate comparisons, AUC values were normalized to those of a control group, allowing for a direct assessment of treatment effects by expressing the AUC of each condition relative to the control. This normalization process ensures that baseline variations are accounted for, providing a clear basis for evaluating how different concentrations impact synaptogenesis. By analyzing these normalized AUC values, researchers can effectively compare the extent of synaptic development across different treatments, offering a robust quantitative framework for ranking substances based on their efficacy in promoting or inhibiting synaptic growth and development.

### 2.8. Calculation of Benchmark Dose

Benchmark concentrations at 10% (BMC 10%) and their lower confidence limits (BMCL) were estimated using PROASTweb software (version 70.1; https://proastweb.rivm.nl/, accessed on 3 October 2024). The software processes assay data, including PFAS concentration levels and their effects, by fitting the data to a model using maximum likelihood estimation (MLE), optimized according to the lowest Akaike Information Criterion (AIC). The AIC is used to select the most appropriate model for the data. The BMC was calculated with a 95% confidence interval and a *p*-value threshold of 0.05, based on the model with the lowest AIC. The BMC and BMCL values, derived from the synaptogenesis assay, represent the point at which significant changes of 10% occur in response rates compared to the control occur, allowing for the ranking of PFAS compounds according to their impact on the neuronal system.

### 2.9. High-Throughput Screening Platform for Behavior

To perform high-throughput behavior assays, we employed the WormLab system (Version 2023.1.1, MBF Bioscience, VT, USA) in conjunction with the COPAS BIOSORT (Union Biometrica, Inc., MA, USA) as previously described [34,35]. Age-synchronized L1-stage nematodes were allocated into individual wells of 96-well plates, each containing 100 µL of the varying concentrations of PFAS along with k-medium and a bacterial food source. The worms were exposed to varying concentrations of individual PFAS compounds. The experiments were conducted in triplicate, with three separate plates, each containing 10 technical replicates per exposure level. Negative controls were included on each plate. The plates were incubated at 25 °C for either 24 or 48 h with continuous shaking. After incubation, the worms were transferred to NGM plates and allowed to acclimate for 1 h before being analyzed. The WormLab system was used to analyze behavioral endpoints, recording the worms’ movement for 60 s with a minimum track duration of 30 s. Images were captured using a Nikon DSLR camera (Tokyo, Japan) at a resolution of 1280 × 960 pixels and a frame rate of 7.5 frames per second. Center point speed was asse, ssed as an indicator of locomotion and overall motor function in *C. elegans.* The nematodes’ movement was tracked to evaluate their speed and coordination, reflecting their neural and muscular integrity [59]. Efficient movement is crucial for the worms’ ability to respond to environmental changes and avoid unfavorable conditions [60]. Data on center point speed were collected, analyzed, and visualized using GraphPad Prism.

### 2.10. Correlation Analysis Between Toxicity and Neurodevelopment

The investigation into the connection between PFAS toxicity and its effects on neurodevelopment sought to clarify how varying levels of toxicity can impact the development of neural structures and functions. To quantify this relationship in *C. elegans*, Pearson correlation analysis was performed (*p* < 0.05), allowing for a detailed assessment of the effects. The findings were illustrated using a heatmap, which visually represented the intricate patterns of interaction between toxicity and neurodevelopmental outcomes. These correlations shed light on the underlying mechanisms that may link PFAS exposure to disruptions in neurodevelopment. For the analysis, the “mice” package in R was utilized to compute the correlation coefficients, enabling a robust statistical evaluation of the data. This comprehensive approach not only enhances our understanding of PFAS’s impact on neurodevelopment but also highlights the significance of examining toxicity in relation to cognitive processes.

### 2.11. Statistical Analysis

Data analysis was performed using GraphPad Prism or R version 3.3.4, and the results were reported as means ± standard deviation (SD). Statistical comparisons between experimental groups and controls were conducted using analysis of variance (ANOVA) to determine overall differences, with subsequent Tukey’s post hoc test applied for pairwise comparisons to identify specific group differences. Significance levels are indicated in the graphs with asterisks (*, *p* ≤ 0.05; **, *p* ≤ 0.01; ***, *p* ≤ 0.001).

## 3. Results

### 3.1. Dopaminergic Neurons

**Figure 2:**
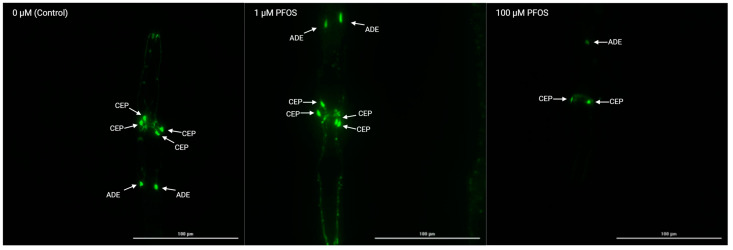
Impacts of PFOS on dopaminergic neurons on BZ555 (dat-1p::GFP) *C. elegans* after 48 h of exposure utilizing the Cytation5 Imaging Multi-Mode Reader at 60× magnification. CEP: Cephalic Sensilla Neurons. ADE: Anterior Deirids Neurons.

To evaluate the developmental toxicity of PFAS, synchronized L1-stage worms were subjected to different concentrations: 100, 1, and 0 μmol/L. As seen in Figure 2 and Figure S1, a notable decrease in fluorescence intensity of dopaminergic neurons (CEP: Cephalic Sensilla Neurons and ADE: Anterior Deirids Neurons) can be visually observed with increasing concentrations of PFOS after 48 h of exposure, highlighting the compound’s impact on neuronal morphological alterations. Additionally, the presence of blebs in images serves as a morphological marker of neurodegeneration and is associated with impaired neuronal function. However, visual observations alone cannot be quantified, necessitating further quantitative analysis to accurately assess the extent of the fluorescence changes. The fluorescence intensity and size of individual dopaminergic neurons was measured at different time points, including 24 and 48 h, using deep learning techniques to analyze and quantify the size and intensity levels. This approach provides a quantitative assessment of the dopaminergic neurons and enables precise measurement of the effects of PFAS on neuronal morphology, facilitating the identification of dose-dependent changes and the comparison of toxicity among different compounds.

**Figure 3:**
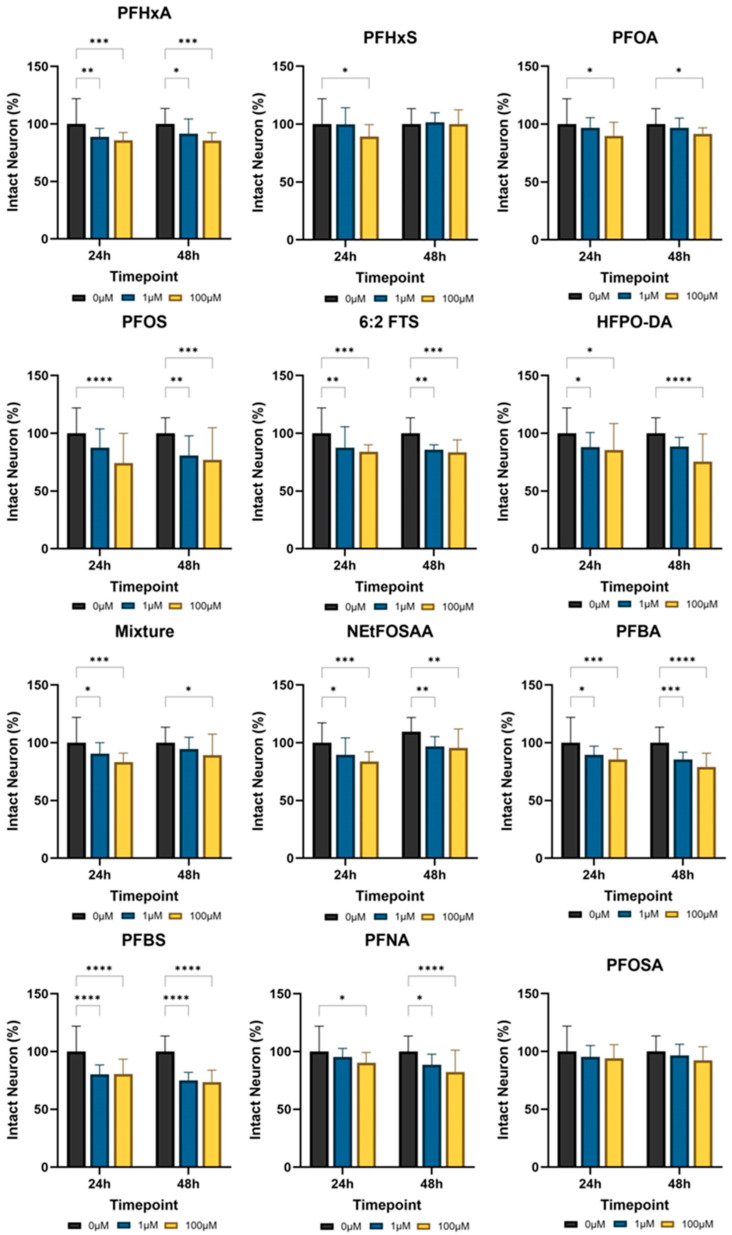
Effects of PFAS on dopaminergic neurons on BZ555 (dat-1p::GFP) *C. elegans* after exposure. All values are represented as percent of intact neuron (*n* = 30, *, *p* < 0.05; **, *p* < 0.01; ***, *p* < 0.001; ****, *p* < 0.0001).

As illustrated in Figure 3, the quantitative fluorescence measurements determined through deep learning allow for a detailed comparison of dopaminergic neuron across various PFAS concentrations. Among the PFAS tested, 6:2 FTS, HFPO-DA, PFBA, PFBS, PFHxA, NEtFOSAA, and PFOS were identified as the most toxic, as they consistently caused significant reductions in intact neurons at both 24 and 48 h, indicating a substantial and immediate impact on dopaminergic neuron morphology. In contrast, PFOA and PFNA only showed significant toxic effects on neurons only at high concentration.

The highest levels of inhibitions were 26.61% for 6:2 FTS, 24.58% for HFPO-DA, 20.90% for PFBA, 26.44% for PFBS, 15.53% for PFHxA, and 25.81% for PFOS. In contrast, PFHxS showed significant decreases in intact neurons at only one of the measured time points, suggesting variations in the rate at which these compounds exert their effects or their ability to induce delayed cellular responses. This temporal specificity highlights that the mechanisms of action for these PFAS compounds may differ, potentially involving complex interactions with neuronal processes that manifest at different stages. Notably, PFOSA did not exhibit any significant decreases in neuronal morphology, indicating a lower or negligible impact on dopaminergic neurons compared to the other PFAS compounds tested.

### 3.2. Synaptogenesis

To examine the developmental toxicity of PFAS, synchronized L1-stage organisms were exposed to a range of concentrations: 200, 100, 10, 1, and 0.1 μmol/L. Synapse formation was assessed using an aldicarb-sensitive assay, measuring the time to paralysis upon aldicarb exposure at 24 and 48 h. The area under the curve (AUC) for paralysis was calculated to quantify the synaptic response, with detailed values of BMD provided in Table 1. This assessment offered insights into how different concentrations affect synaptic development and enabled a comparison of the efficacy of various test substances in modulating synaptogenesis.

**Figure 4:**
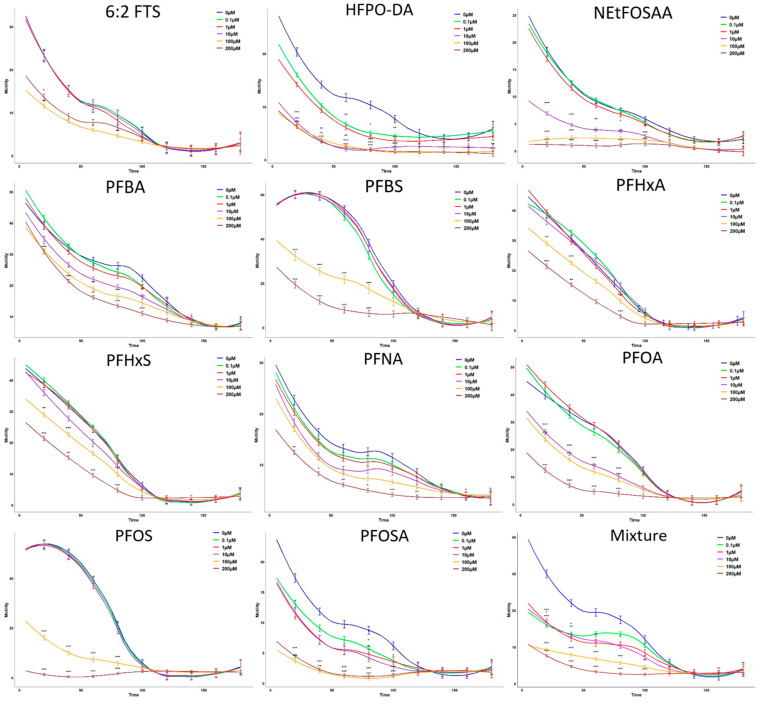
Impacts of PFAS on synaptogenesis of *C. elegans* after 48 h of exposure. All values are represented as motility (*n* = 30, *, *p* < 0.05; **, *p* < 0.01; ***, *p* < 0.001).

As shown in Figure 4, the aldicarb-induced paralysis assay revealed significant alterations in synaptogenesis across different concentrations at 48 h. The most pronounced effects on synaptic transmission were observed at concentrations of 100–200 μmol/L, where there was a notable decrease in the time to paralysis, indicating enhanced disruption to synaptic function (Figure 4 and Figure S2). Specifically, PFOS and PFBS exhibited significant changes in synaptic response, as evidenced by a marked reduction in motility and increased time to paralysis at both 24 and 48 h. This suggests that these compounds strongly impact synaptic transmission over time. The assay demonstrated that higher concentrations of these PFAS compounds lead to more severe impairments in synaptic activity, highlighting their potent effects on neurotransmission. The maximum inhibition rates were 45.7% for 6:2 FTS, 73.8% for HFPO-DA, 91.4% for NEtFOSAA, 33.2% for PFBA, 72.2% for PFBS, 48.6% for PFHxA, 48.5% for PFHxS, 72.8% for PFOA, 88.9% for PFOS, 48.2%for PFNA, 71.9% for PFOSA, and 72.3% for the mixture. According to the BMC value, the toxicity ranks of ten PFAS in the adult stage were as follows, with lower BMC values indicating higher toxicity: PFBA > NEtFOSAA > HFPO-DA > PFOSA > Mixture > PFOS > 6:2 FTS > PFHxS > PFOA > PFHxA > PFNA > PFBS.

### 3.3. Behavior

To investigate the effects of PFAS on behavioral performance, synchronized L1-stage *C. elegans* were exposed to varying concentrations of PFAS: 200, 100, 10, 1, and 0.1 μmol/L. Center point speed was measured to evaluate the impact on locomotion and motor function. This behavioral assessment was conducted at 24 and 48 h post-exposure. Changes in center point speed provided insights into how different PFAS concentrations affect the worms’ ability to move and respond to their environment. Detailed results, including the benchmark concentration of the mean center point speed for each concentration, are presented in Table 2. This analysis allowed for a comparative evaluation of the effects of various PFAS on nematode locomotion and motor coordination.

**Figure 5:**
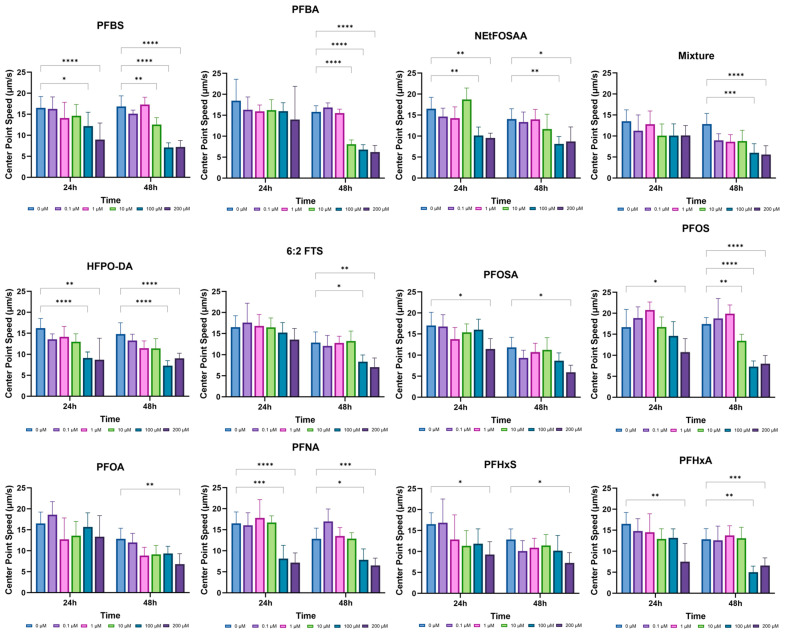
Effects of PFAS on behavior (center point speed) on N2 (wild type) *C. elegans* after exposure. All values are represented as center point speed (µm/s) (*n* = 30, *, *p* < 0.05; **, *p* < 0.01; ***, *p* < 0.001; ****, *p* < 0.0001).

As illustrated in Figure 5, the assessment of center point speed revealed significant effects on locomotion across different PFAS concentrations. The most notable reductions in speed were observed at concentrations of 100–200 μmol/L, indicating a marked impairment in the worms’ movement ability (Figure 5). Specifically, PFOS and PFBS caused substantial decreases in center point speed, with pronounced effects evident at both 24 and 48 h. This indicates that these compounds have a strong influence on nematode motility over time. The analysis demonstrated that higher concentrations of these PFAS compounds lead to greater reductions in center point speed, underscoring their significant impact on locomotor function. The maximum reductions in speed were observed with PFOS, PFBS, and PFNA, with the highest inhibition rates at 54.13% for PFOS, 57.06% for PFBS, and 49.31% for PFNA. According to the BMC value, the toxicity ranks of ten PFAS in the adult stage were as follows: Mixture > HFPO-DA > PFBA > PFOS > NEtFOSAA > PFBS > 6:2 FTS > PFNA > PFHxA > PFHxS > PFOSA > PFOA.

### 3.4. Correlation Analysis Between Toxicity and Neurodevelopment

**Figure 6:**
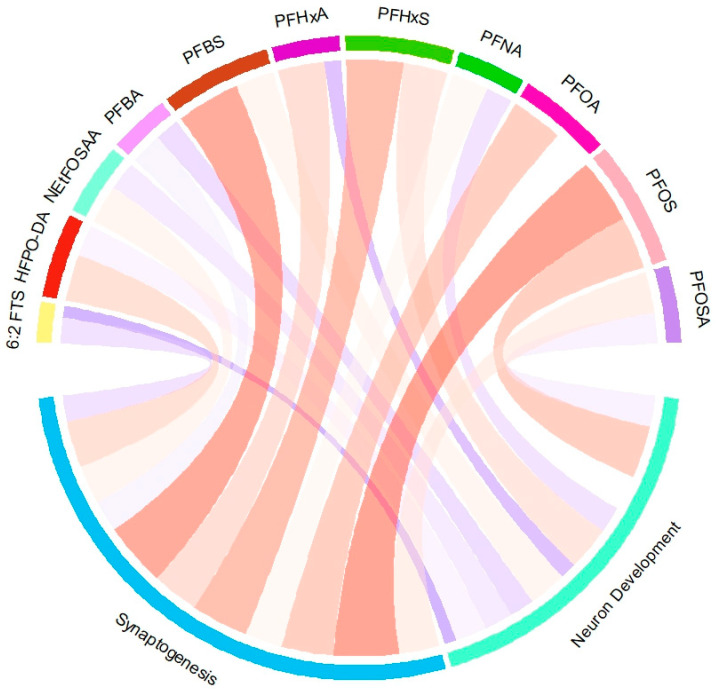
Correlation between toxicity and neurodevelopment: blue indicates positive and red indicates negative correlations. All values are represented as a Pearson Correlation Coefficient. The correlation circle was plotted using the “chord” package in R (version 3.3.4).

To assess the relationship between PFAS toxicity and its effects on neurodevelopment and synaptogenesis, a comprehensive analysis was conducted using the Pearson Correlation Coefficient, calculated through the “mice” package in R software (The R Foundation, Vienna, AUT, version 3.3.4). This evaluation took into account both the behavioral toxicity and the impacts on neural development processes in addition to the synaptogenesis observed after 48 h of exposure to various PFAS compounds. These results, illustrated in Figure 6, revealed distinct correlations between PFAS toxicity and neurodevelopment. PFOS, PFBS, and PFHxS exhibited the strongest negative correlations with neurodevelopment and synaptogenesis, indicating that these substances are particularly harmful and may inhibit normal neural growth and function. In contrast, 6:2 FTS and PFHxA showed minimal correlation, which may reflect differences in their mechanisms of action, despite both chemicals affecting neurodevelopment in the assays.

## 4. Discussion

In this study, individual PFAs and a reference mixture were selected to examine the impacts on neurodevelopment and the neuronal system in *C. elegans*. A high-throughput, high-content screening platform, enhanced by AI, was employed for this comprehensive assessment. Our findings revealed that PFAS significantly hindered the neurodevelopment of *C. elegans* at concentrations ranging from 100 to 200 µM after 48 h of exposure during their developmental stages. Our results demonstrated that 6:2 FTS, HFPO-DA, PFBA, PFBS, PFHxA, and PFOS significantly inhibited dopaminergic neuron activity in synchronized L1-stage worms, with notable reductions in size and fluorescence intensity observed across a concentration range of 1 to 100 μmol/L. This decline in neuronal fluorescence indicates a dose-dependent impact of these PFAS compounds on neuronal health, with PFOS and PFBS showing the most significant effects.

Further analysis through aldicarb-induced paralysis assays revealed that PFAS not only affected dopaminergic neurons but also significantly disrupted synaptic transmission. This disruption was characterized by decreased motility and increased paralysis times, particularly at higher concentrations, indicating a profound impact on the worms’ neuromuscular function as seen in the evaluation of behavior. Although both neuron-specific inhibition and synaptic inhibition were observed, the slight differences in their effects suggest that these processes may be influenced by similar but slightly divergent mechanisms. These nuances in how PFAS compounds interact with neuronal and synaptic functions underscore the complexity of their toxicity. Among the tested PFAS, PFOS emerged as one of the most potent, exhibiting significant toxicity across all neurodevelopmental endpoints.

The widespread occurrence of PFAS in human populations, coupled with evidence of potential adverse effects, has led to growing apprehension about the possible implications of the abundant usage [61]. In response to findings from dose-dependent toxicity studies, epidemiologists have increasingly concentrated on investigating legacy PFAS, such as PFOS and PFOA, and their impact on human adverse effects [62]. However, due to the bioaccumulation nature of PFAS, there has been increasing international regulatory efforts on legacy compounds [63]. In response to regulations, alternative PFAS have been introduced to obtain similar goals [64]. A majority of the alternative PFAS are not well-documented or identified due to their nature as proprietary compounds or by products of manufacturing processes [65]. In the present study, we identified eleven PFAS from the most commonly found compounds in major U.S. water systems [48]. Additionally, a reference mixture was prepared from the identified compounds, employing their observed ratios to facilitate the simultaneous testing of multiple substances. We observed that the toxicity of the mixture differed from the individual components. The mixture had a higher concentration than some of the individual PFAS, potentially leading to interactions between the substances that may have influenced the overall toxicity. However, this requires further investigation to fully understand the underlying mechanisms.

The nematode, *Caenorhabditis elegans*, has been an invaluable model organism in scientific research for almost fifty years [66]. *C. elegans* offers numerous advantages as a model organism, including its ease of genetic manipulation, small size, and low-cost maintenance, while its transparent body enables the direct visualization of fluorescently labeled cells [67]. *C. elegans* have been extensively studied for in vivo toxicity to assess how exposure affects individual cells and organs [68]. *C. elegans* possesses four key organ systems—neural, digestive, immune, and reproductive—that are analogous to those found in vertebrates, making its findings both reliable and significant for comparative studies [69]. Employing high-throughput, high-content screening platform developed in our laboratory [35,70], the neurodevelopmental toxicity of PFAS was assessed by analyzing their impact on individual neurons and the entire neural network, taking into account localized as well as systemic effects. The neuropeptide signaling in nematodes, despite their limited number of neurons and simpler anatomy, shows an unexpected level of conservation and diversity similar to the signaling observed in the human brain [71]. The findings from this part of the study were in agreement with previous research, revealing that most PFAS and their mixtures had detrimental effects on both neuronal development and synaptic transmission [33]. While limited to only PFOS, the previous research laid the foundation for further investigation into PFAS.

Furthermore, the investigation of dopaminergic (DA) neurons is critical for understanding neurotoxic impacts because these neurons are highly involved in controlling movement, mood regulation, and reward mechanisms, all of which are crucial for proper neurological function [72]. Disruptions to DA neuron activity are known to be associated with neurodevelopmental disorders, including Parkinson’s disease and other neurodegenerative conditions [73]. As such, studying PFAS compounds effect on DA neurons can provide valuable insights into their potential role in the development of neurological diseases. Furthermore, *C. elegans* provides an ideal model for such investigations due to its well-characterized dopaminergic system and the ability to directly observe changes in DA neuron function, morphology, and behavior. Dopaminergic (DA) neurons play a pivotal role in neurodevelopment, as they are involved in essential processes such as neuronal differentiation, synaptic formation, and the establishment of neural circuits, making them critical targets for studying developmental neurotoxicity [74].

Building on this groundwork, our research is crucial as it expands the scope by including a broader array of PFAS compounds and their mixtures, allowing us to explore their diverse effects on dopaminergic (DA) neurons, neurodevelopment, and overall health. By examining additional PFAS, we aim to provide a more comprehensive understanding of their neurotoxic effects and environmental impact, addressing gaps left by earlier studies and contributing valuable insights for regulatory and public health considerations. Previous studies in mammalian and non-mammalian models have shown that PFAS disrupt crucial neurotoxic targets for neurotransmission [27]. These studies have demonstrated that PFAS disrupt neurotransmitter levels [75], interfere with the expression and function of synaptic proteins [76], and alter calcium signaling pathways [77]. By employing a diverse range of model organisms, researchers have gained deeper insights into how PFAS influence neural communication and signaling, particularly with regard to DA neurons. These findings underscore the need for further investigation into how PFAS disrupt these essential neural circuits. Although there is extensive research on PFAS in mammalian models, these studies have limitations, and many PFAS chemicals still lack mammalian data [78]. Additionally these studies come with significant drawbacks, including ethical concerns, high expenses, extended study durations due to the long lifespans of the animals, and limited sample sizes, which can pose challenges for achieving statistically significant results [79]. In contrast, *C. elegans* present a cost-effective and efficient alternative offering a practical solution for studying PFAS neurodevelopmental toxicity, with the added advantage of a well-characterized dopaminergic system, making it an ideal platform for examining the impact of PFAS on DA neuron function and overall neurodevelopment.

As experimental platforms continue to generate increasingly large and complex datasets, there is a rising need for real-time processing and high-performance computing to manage and analyze the vast amounts of data being produced [80]. This growing reliance on advanced computing technologies is essential for efficiently storing, processing, and interpreting data at the high speeds required by modern research. The growth of AI technology and its utilization is transforming how we analyze various datasets, greatly enhancing our ability to explore and understand intricate biological systems. High-throughput, high-content screening platforms often generate extensive datasets, including high-content imaging data [81]. Despite technological advances, the visual inspection of phenotypes and tissue features remains a fundamental method. Typically, this involves manually reviewing microscopic images to identify and differentiate between normal and abnormal phenotypic characteristics [82]. However, manual investigation of image datasets comes with several limitations. As the volume of data increases, detecting subtle changes in morphology and performing detailed quantitative evaluations becomes progressively more difficult, even for seasoned professionals. Previous research has utilized AI-based image analysis to investigate neural network development in zebrafish [83]. Leveraging AI for image analysis in *C. elegans* neural network studies can deliver more detailed and extensive insights into the impacts of neurotoxins. Furthermore, these programs can lead to significant advancements in neurodevelopment and environmental toxicology research.

The combination of a nematode model with sophisticated high-throughput, high-content screening coupled with AI technology enhances the capability to study PFAS toxicity and its impact on neurodevelopment. These results are poised to advance our comprehension of PFAS-induced neurodevelopmental toxicity by addressing critical gaps in current knowledge. Specifically, this study examines the impacts of PFAS on the dopaminergic neuron morphological alterations, synaptic formation, and neuron activity. However, a limitation of this study is the limited range of exposure levels assessed, which restricts a more comprehensive evaluation of the impacts on neuronal development. Understanding the mechanisms behind PFAS toxicity is crucial for refining risk assessments and safety evaluations related to these substances. Additionally, it is important to recognize that individuals are frequently exposed to multiple PFAS simultaneously from diverse sources, with the specific mixtures and concentrations varying significantly by geographical region. This variability underscores the complexity of PFAS exposure and the need for thorough investigation into its cumulative effects of several combinations. Although evaluating every possible PFAS combination is unfeasible, it is crucial to conduct research that reflects real-world exposure scenarios, specifically in high-population areas, to better understand developmental neurotoxicity.

## 5. Conclusions

By employing a high-throughput, high-content screening approach integrated with AI, we assessed the effects of individual PFAS and a reference mixture on both neurodevelopment and the neuronal system. Our findings indicate that PFAS have a detrimental impact on neurodevelopment in C. elegans. This study found that PFAS compounds with higher toxicity in our assays were associated with more significant neuronal damage, although the relationship with mammalian toxicity remains to be explored. Additionally, exposure to PFAS mixtures appears to exacerbate neurodevelopmental issues. These results provide crucial insights into the potential harmful effects of PFAS exposure and underscore the need for comprehensive studies on developmental exposure.

## Figures and Tables

**Table 1:** The benchmark concentration 10% PFAS on synaptogenesis at different time points.

PFAS	Time Point	PROAST BEST Model	BMC (BMCL) [µM]
6:2 FTS	24 h	Expon. m3-	32.28 (21.20)
	48 h	Hill m5-	23.21 (13.30)
HFPO-DA	24 h	Expon. m5-	4.96 (2.58)
	48 h	Expon. m5-	0.62 (0.26)
Mixture	24 h	Expon. m3-	26.30 (17.40)
	48 h	Hill m3-	5.17 (1.94)
NEtFOSAA	24 h	Expon. m5-	0.65 (0.15)
	48 h	Expon. m5-	1.36 (0.11)
PFBA	24 h	Expon. m5-	0.22 (0.0065)
	48 h	Hill m3-	0.40 (0.011)
PFBS	24 h	Expon. m5-	52.39 (8.17)
	48 h	Hill m5-	45.25 (9.33)
PFHxA	24 h	Hill m3-	0.83 (0.00016)
	48 h	Expon. m3-	37.00 (21.20)
PFHxS	24 h	Expon. m3-	111.40 (70.10)
	48 h	Expon. m3-	34.93 (24.90)
PFNA	24 h	Expon. m3-	99.77 (70.20)
	48 h	Expon. m3-	40.12 (11.20)
PFOA	24 h	Hill m3-	0.059 (0.000048)
	48 h	Expon. m3-	35.80 (15.90)
PFOS	24 h	Expon. m3-	20.37 (11.90)
	48 h	Expon. m3-	17.56 (10.80)
PFOSA	24 h	Expon. m3-	0.44 (0.022)
	48 h	Expon. m5-	10.14 (4.81)

BMC: benchmark concentration; BMCL; benchmark concentration lower-confidence limit.

**Table 2:** The benchmark concentration 10% PFAS on behavior (center point speed) at different time points.

PFAS	Time Point	PROAST BEST Model	BMC (BMCL) [µM]
6:2 FTS	48 h	Expon. m3-	11.51 (3.42)
HFPO-DA	24 h	Expon. m3-	0.89 (0.0080)
	48 h	Expon. m5-	0.22 (0.13)
Mixture	48 h	Hill m3-	0.0043 (0.000024)
NEtFOSAA	24 h	Expon. m5-	50.59 (12.70)
	48 h	Hill m5-	5.87 (0.67)
PFBA	24 h	Hill m3-	122.70 (84.40)
	48 h	Hill m5-	0.72 (0.16)
PFBS	24 h	Expon. m3-	41.63 (9.00)
	48 h	Expon. m5-	8.03 (1.51)
PFHxA	24 h	Expon. m3-	94.12 (59.50)
	48 h	Expon. m5-	41.7 (7.57)
PFHxS	24 h	Expon. m5-	0.073 (0.000065)
	48 h	Expon. m3-	48.55 (14.10)
PFNA	24 h	Hill m5-	32.04 (13.90)
	48 h	Expon. m5-	28.79 (6.72)
PFOA	48 h	Expon. m3-	82.48 (41.10)
PFOS	24 h	Expon. m3-	29.4 (7.12)
	48 h	Hill m5-	4.62 (1.21)
PFOSA	24 h	Hill m3-	148.3 (103.00)
	48 h	Expon. m3-	56.56 (8.14)

BMC: benchmark concentration; BMCL; benchmark concentration lower-confidence limit.

## Data Availability

The original contributions presented in this study are included in the article/Supplementary Material. Further inquiries can be directed to the corresponding author.

## Supplementary Materials

The following supporting information can be downloaded at: https://www.mdpi.com/article/10.3390/jox15010024/s1. Figure S1: Impacts of PFAS on dopaminergic neurons on BZ555 (dat-1p::GFP) *C. elegans* after 48 h of exposure utilizing the Cytation5 Imaging Multi-Mode Reader at 60× magnification. CEP: Cephalic Sensilla Neurons. ADE: Anterior Deirids Neurons.; Figure S2: Impacts of PFAS on synaptogenesis of *C. elegans* after 24 h of exposure. All values are represented as motility.

## Abbreviations

The following abbreviations are used in this manuscript:

AI	Artificial intelligence
PFAS	Per- and polyfluoroalkyl substances
PFBA	Perfluorobutanoic acid
PFHxA	Perfluorohexanoic acid
PFOA	Perfluorooctanoic acid
PFNA	Perfluorononanoic acid
PFBS	Perfluorobutanesulfonic acid
PFHxS	Perfluorohexanesulphonic acid
PFOS	Perfluorooctanesulfonic acid
PFOSA	Perfluorooctanesulfonamide
NEtFOSAA	N-ethyl perfluorooctanesulfonamidoacetic acid
6:2 FTS	6:2 fluorotelomer sulfonic acid
HFPO-DA	Hexafluoropropylene oxide dimer acid
DNT	Developmental neurotoxicity
ASD	Autism spectrum disorder
ADHD	Attention-deficit hyperactivity disorder
DA	Dopaminergic
NGM	Nematode growth media
CNN	Convolutional neural network
AUC	Area under the curve
BMC	Benchmark concentration
BMCL	Benchmark concentration lower-confidence limit
