# Supplementary material for: Utilization of Artificial Intelligence Coupled with a High-Throughput, High-Content Platform in the Exploration of Neurodevelopmental Toxicity of Individual and Combined PFAS
Source: J Xenobiot. 2025 Feb 2;15(1):24. doi: 10.3390/jox15010024 (PMC11857074; doi:10.3390/jox15010024)

Figure S1: Impacts of PFAS on Dopaminergic Neurons on BZ555 (dat-1p::GFP) *C. elegans* after 48 hours of exposure utilizing the Cytation5 Imaging Multi-Mode Reader at 60x magnification. CEP: Cephalic Sensilla Neurons. ADE: Anterior Deirids Neurons.

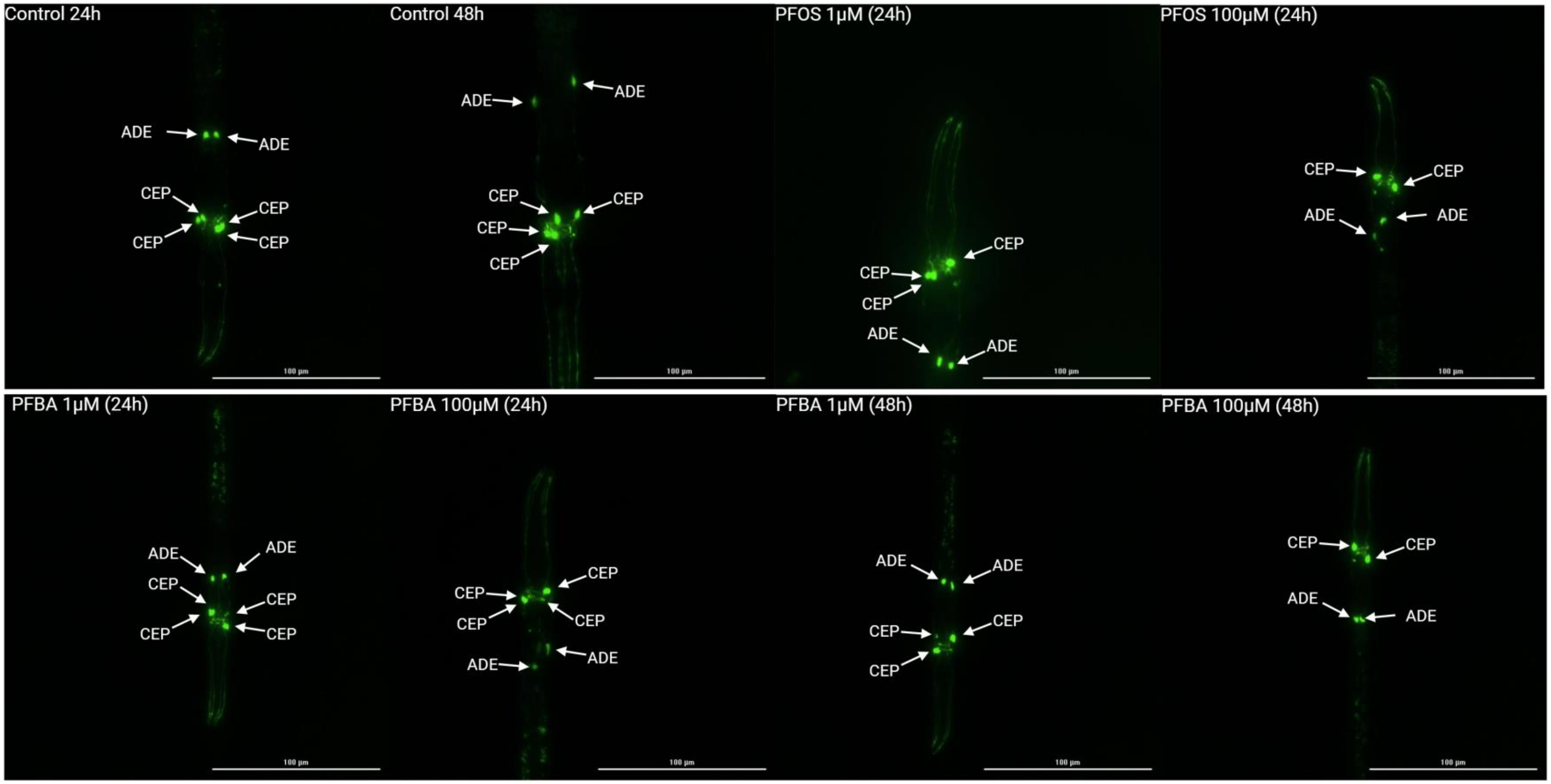

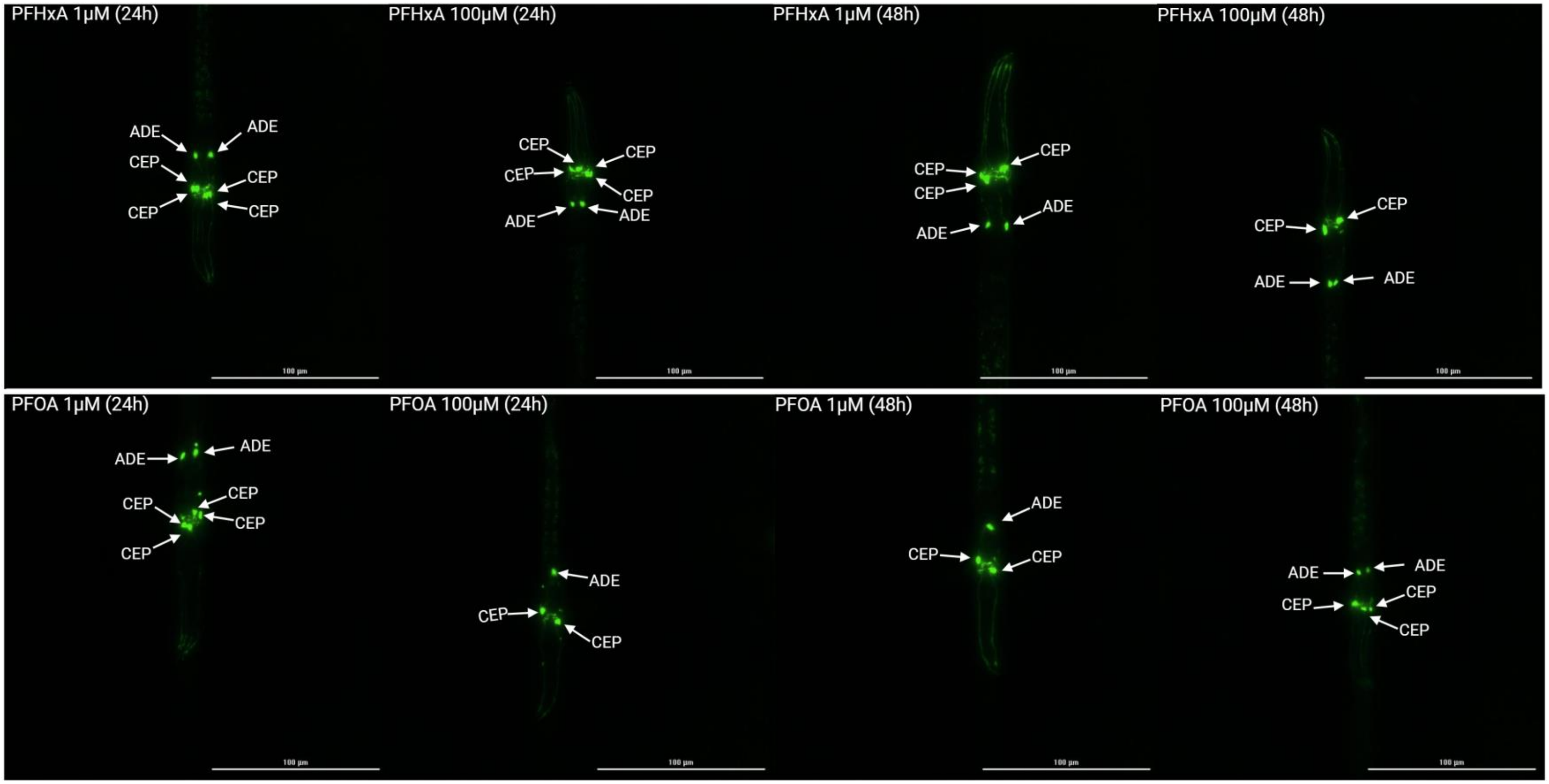
PFHxA 1µM (24h)
ADE
ADE
CEP
CEP
CEP
CEP
PFHxA 100µM (24h)
CEP
CEP
CEP
CEP
ADE
ADE
PFHxA 1µM (48h)
CEP
CEP
CEP
ADE
ADE
PFHxA 100µM (48h)
CEP
CEP
ADE
ADE
PFOA 1µM (24h)
ADE
ADE
CEP
CEP
CEP
CEP
PFOA 100µM (24h)
ADE
CEP
CEP
PFOA 1µM (48h)
ADE
CEP
CEP
PFOA 100µM (48h)
ADE
ADE
CEP
CEP
CEP

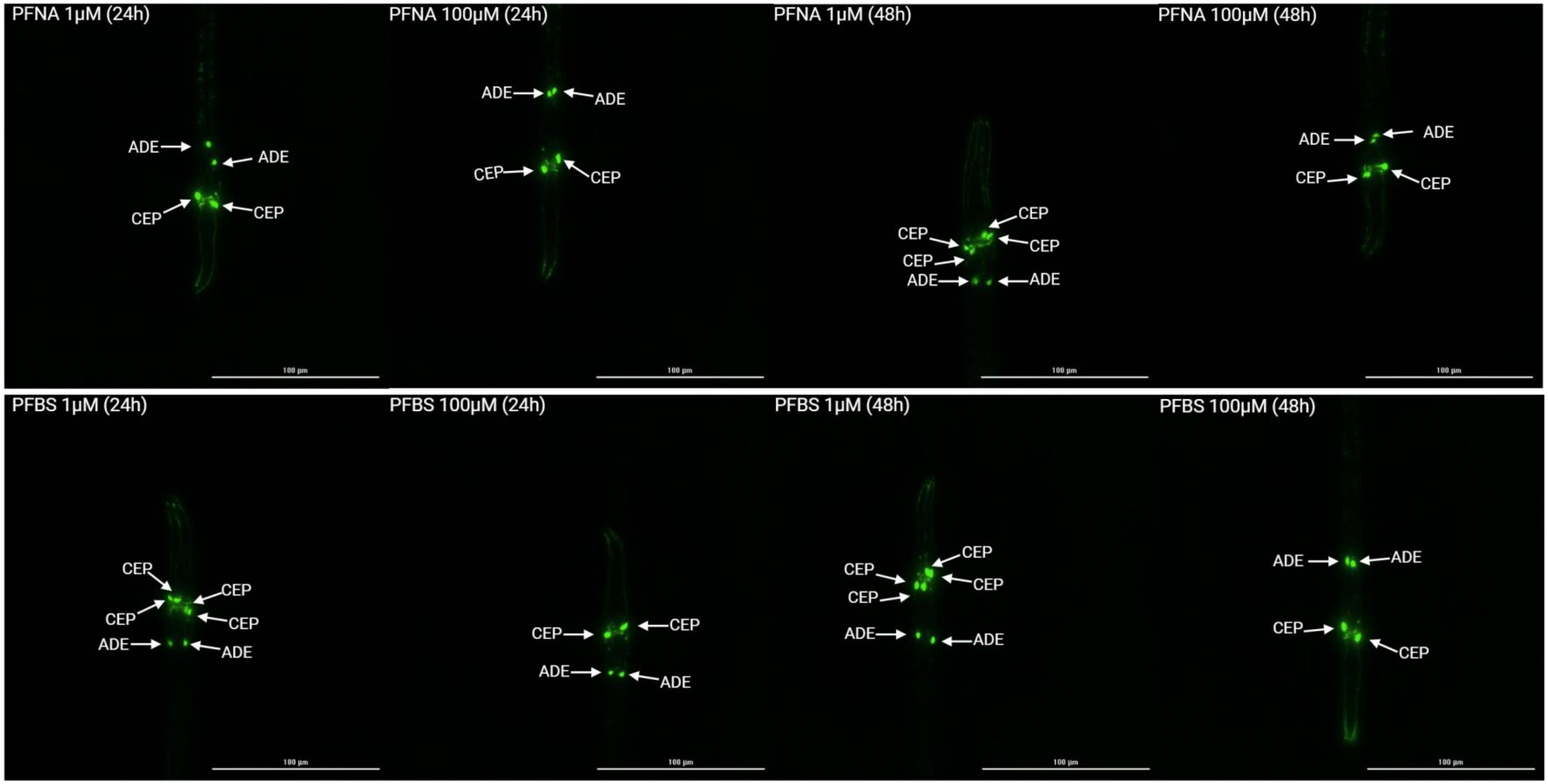
PFNA 1μM (24h)
ADE
ADE
CEP
CEP
PFNA 100μM (24h)
ADE
ADE
CEP
CEP
PFNA 1μM (48h)
CEP
CEP
CEP
CEP
ADE
ADE
PFNA 100μM (48h)
ADE
ADE
CEP
CEP
PFBS 1μM (24h)
CEP
CEP
CEP
CEP
ADE
ADE
PFBS 100μM (24h)
CEP
CEP
ADE
ADE
PFBS 1μM (48h)
CEP
CEP
CEP
CEP
ADE
ADE
PFBS 100μM (48h)
ADE
ADE
CEP
CEP

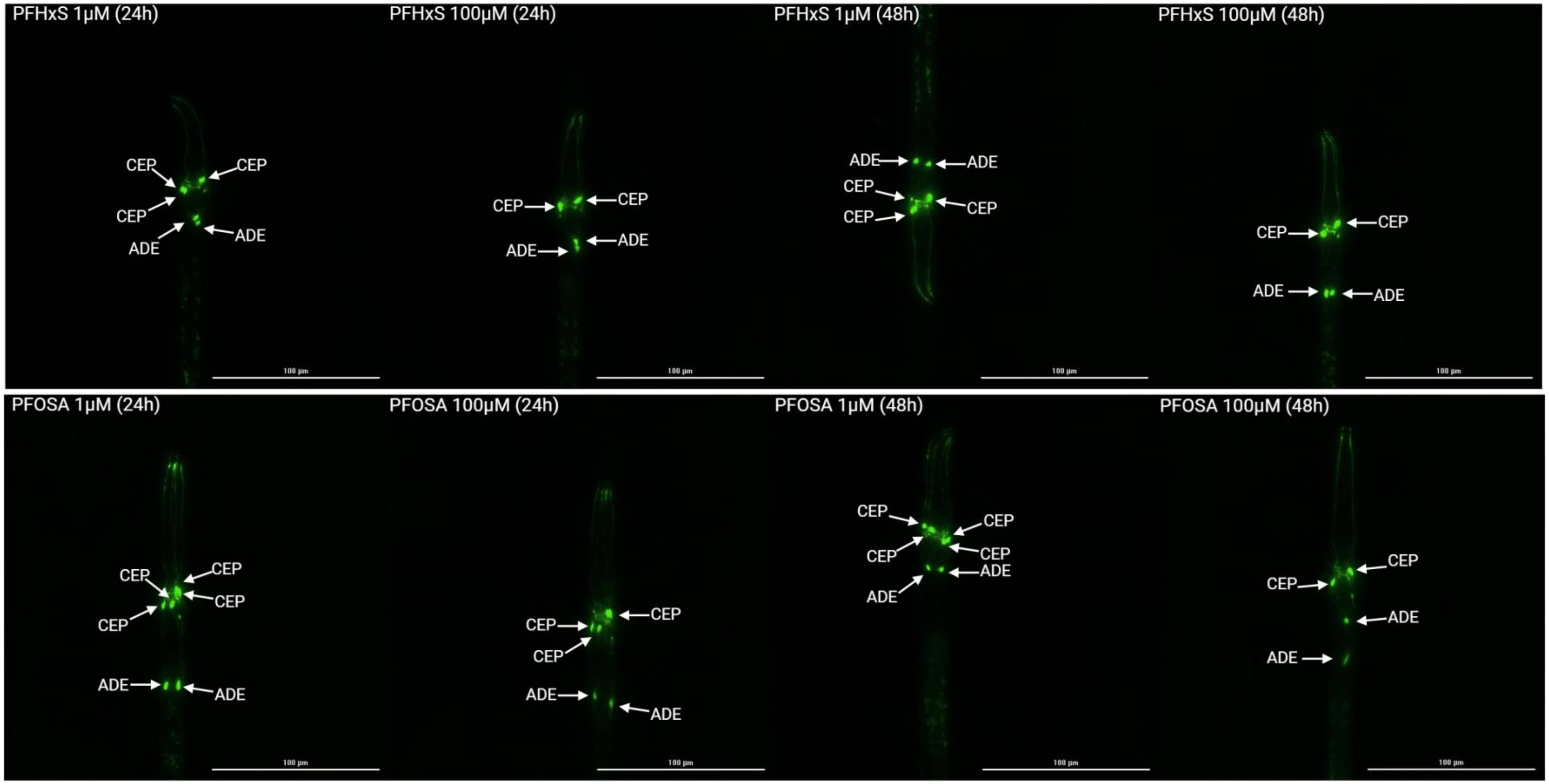
PFHxS 1μM (24h)
PFHxS 100μM (24h)
PFHxS 1μM (48h)
PFHxS 100μM (48h)
PFOSA 1μM (24h)
PFOSA 100μM (24h)
PFOSA 1μM (48h)
PFOSA 100μM (48h)
CEP
ADE

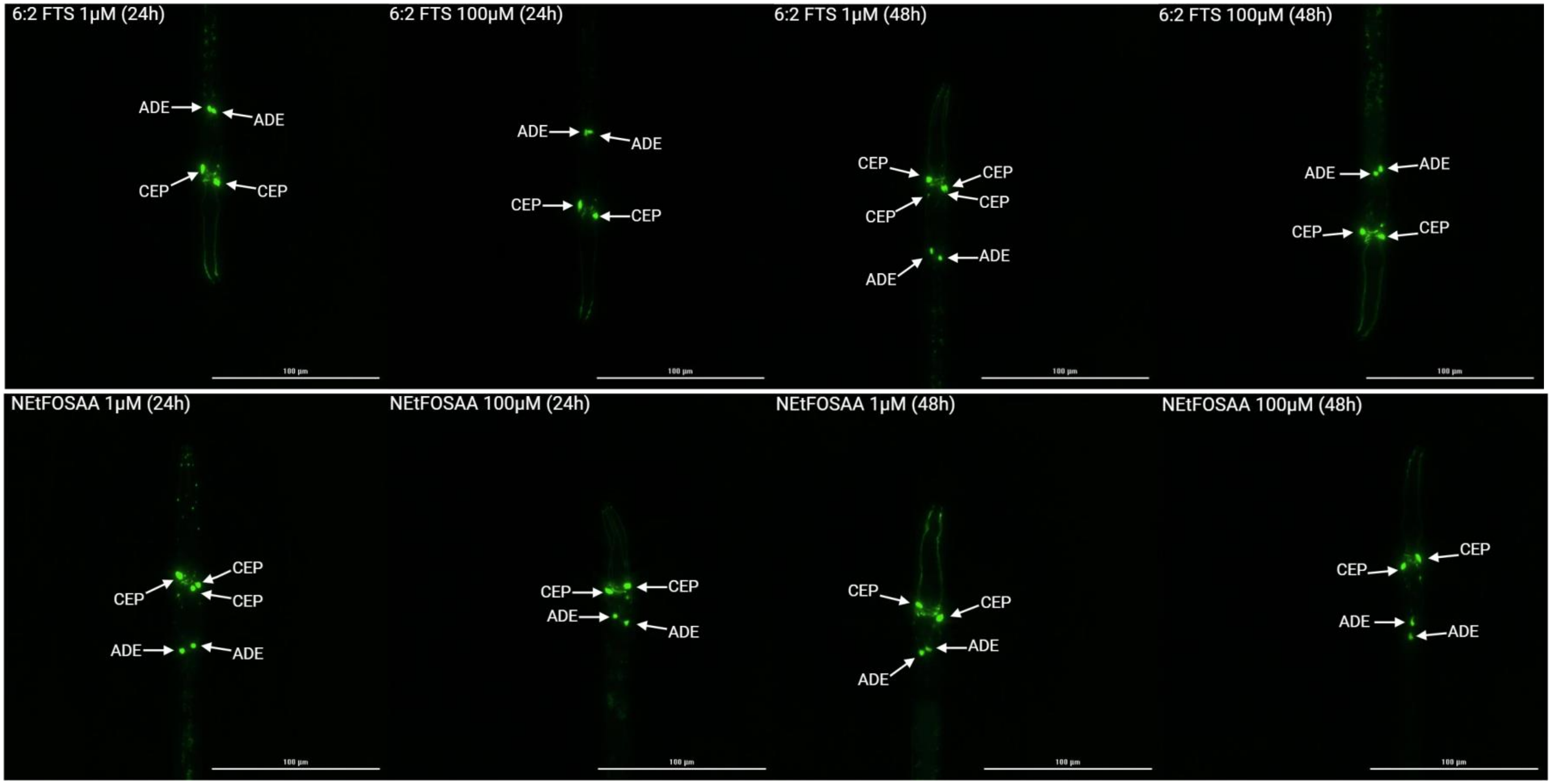
6:2 FTS 1μM (24h)
ADE
ADE
CEP
CEP
6:2 FTS 100μM (24h)
ADE
ADE
CEP
CEP
6:2 FTS 1μM (48h)
CEP
CEP
CEP
CEP
ADE
ADE
6:2 FTS 100μM (48h)
ADE
ADE
CEP
CEP
NEtFOSAA 1μM (24h)
CEP
CEP
CEP
ADE
ADE
NEtFOSAA 100μM (24h)
CEP
CEP
ADE
ADE
NEtFOSAA 1μM (48h)
CEP
CEP
ADE
ADE
NEtFOSAA 100μM (48h)
CEP
CEP
ADE
ADE

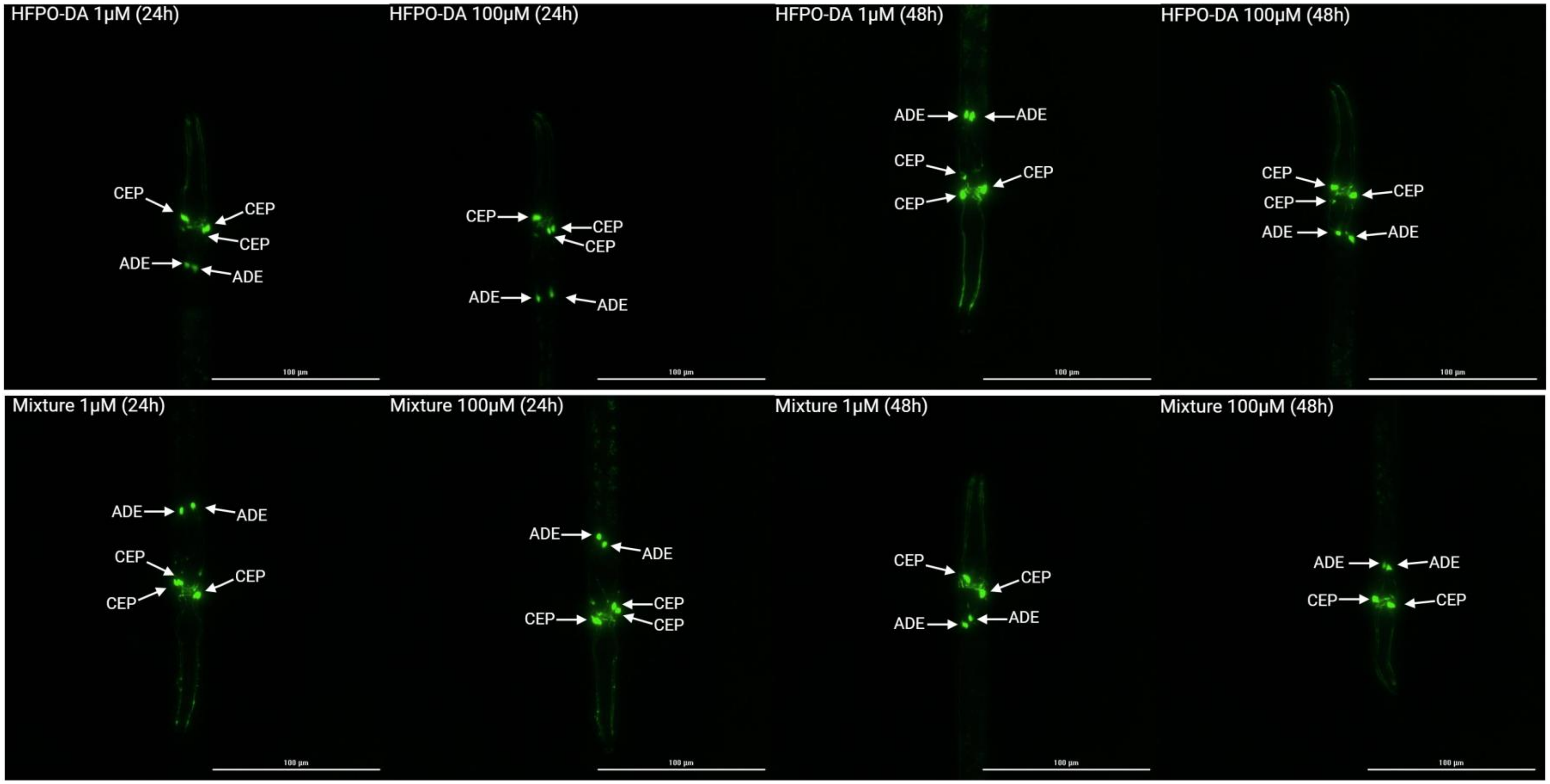

HFPO-DA 1μM (24h)
CEP
CEP
CEP
ADE
ADE
100 μm
HFPO-DA 100μM (24h)
CEP
CEP
CEP
ADE
ADE
100 μm
HFPO-DA 1μM (48h)
ADE
ADE
CEP
CEP
CEP
100 μm
HFPO-DA 100μM (48h)
CEP
CEP
CEP
ADE
ADE
100 μm
Mixture 1μM (24h)
ADE
ADE
CEP
CEP
CEP
100 μm
Mixture 100μM (24h)
ADE
ADE
CEP
CEP
CEP
100 μm
Mixture 1μM (48h)
CEP
CEP
ADE
ADE
100 μm
Mixture 100μM (48h)
ADE
ADE
CEP
CEP
100 μm

Figure S2: Impacts of PFAS on Synaptogenesis of *C. elegans* after 24 hours of exposure. All values are represented as motility.

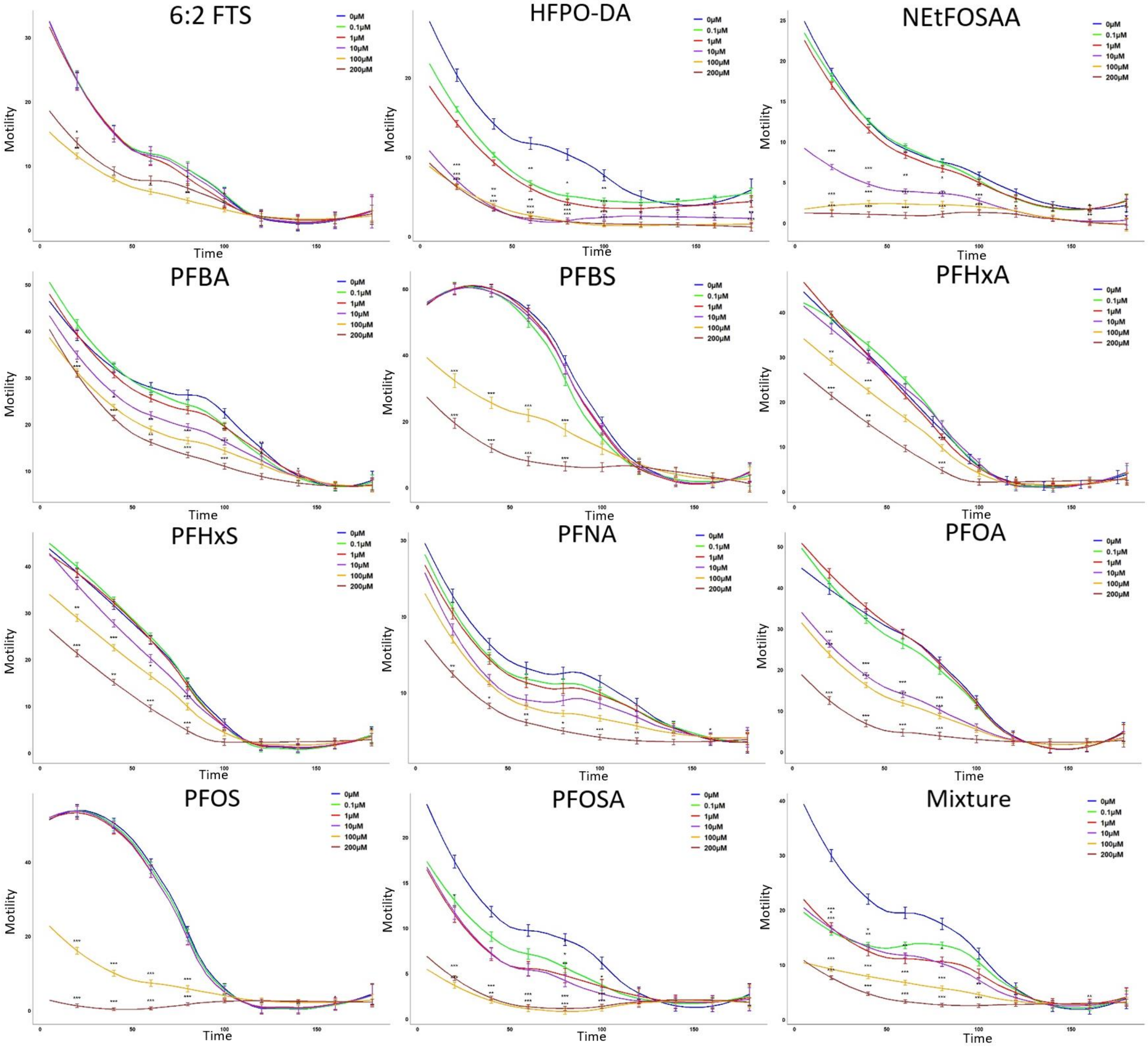

Supplement: Supplementary file 1 [file jox-15-00024-s001.zip › jox-3405550-supplementary.pdf]
